# Predictors of Persistent Pain after Total Knee Arthroplasty

**DOI:** 10.3390/life14101300

**Published:** 2024-10-14

**Authors:** Ali H. Alyami, Mohammed A. Alkhotani, Abdulaziz Abdullah Alsiraihi, Abdulaziz Farouk Y. Bokhari, Mohammed Majed Bukhari, Omar E. Hetta, Hassan O. Bogari, Mohamed Eldigire Ahmed

**Affiliations:** 1 Department of Orthopedic Surgery, Ministry of the National Guard-Health Affairs, Jeddah, Saudi Arabia; alyamia@yahoo.com; 2 King Abdullah International Medical Research Center, Jeddah, Saudi Arabia; 3 King Saud bin Abdulaziz University for Health Sciences, Jeddah, Saudi Arabia; 4 College of Medicine, King Saud bin Abdulaziz University for Health Sciences, Jeddah, Saudi Arabia; 5 College of Science and Health Professions, King Saud Bin Abdulaziz University for Health Sciences, Jeddah, Saudi Arabia

**Keywords:** total knee arthroplasty, persistent postoperative pain, risk factors

## Abstract

Background: Total knee arthroplasty (TKA) is an orthopedic procedure performed on patients with severe knee pain and advanced knee conditions, such as osteoarthritis and rheumatoid arthritis, in order to restore joint function. Despite the procedure’s high success rates, persistent postoperative pain (PPP) remains a significant complication, affecting a substantial proportion of patients. Identifying predictors of PPP is crucial for improving patient outcomes and satisfaction. Methods: A retrospective analytic study was conducted, reviewing the medical records of patients who underwent unilateral or bilateral TKA at King Abdulaziz Medical City. The data collection focused on demographics, comorbidities, clinical presentations, surgical details, and postoperative outcomes. Data were analyzed using JMP software. A *p*-value of less than 0.05 was considered statistically significant. Results: This study included 838 patients, predominantly female (71.5%), with an average age of 65.4 years. Osteoarthritis was the primary reason for surgery (98.3%). The mean preoperative pain score was 3.4, and the average pain duration prior to surgery was 6.2 years. We identified dyslipidemia as a significant predictor of PPP (OR 1.40, *p* = 0.042), while we found younger age to be a significant predictor (OR 0.979, 95% CI 0.967–0.991, *p* = 0.001). Other factors such as gender, diabetes, hypertension, cardiovascular disease, anxiety disorder, mood disorder, tobacco use, chronic kidney disease, chronic lung disease, and BMI were not significant predictors of PPP. Conclusion: This study identifies younger age and dyslipidemia as significant predictors of persistent postoperative pain and improved outcomes following total knee arthroplasty Further research is needed to validate these results in diverse populations and settings, with the objective should be to refine preoperative counseling and postoperative pain management protocols.

## 1. Introduction

Total knee arthroplasty (TKA), also known as total knee replacement, is a surgical intervention aimed at replacing a damaged or deteriorated knee joint with an artificial one, typically crafted from metal and plastic components. This procedure has had a substantial increase in utilization within the orthopedic surgery specialty, with projections indicating a staggering 673% rise in the total number of knee replacement surgeries in the United States, projected to escalate from 0.5 million in 2005 to 3.48 million by the year 2030 [1]. TKA is predominantly recommended for individuals suffering with severe knee pain and impaired mobility attributed to conditions such as osteoarthritis, rheumatoid arthritis, or traumatic injury [2]. Generally considered a treatment of last resort, TKA is reserved for patients with advanced or severe knee damage who have not achieved satisfactory outcomes with other therapeutic modalities like medication, physical therapy, or lifestyle modifications [3]. Despite the procedure’s commendable success rate, several potential complications may arise, with persistent postoperative pain (PPP) emerging as a noteworthy concern for individuals post-TKA [4,5,6,7,8,9].

Persistent postoperative pain (PPP), also known as chronic post-surgical pain, is characterized by pain persisting for more than three months following surgery [10]. Estimates indicate that the prevalence of PPP among TKA patients ranges from 13% to 44% [10]. Various factors have been identified in association with PPP, including intraoperative nerve injury, preoperative pain levels, and psychological variables. This can be seen in a 2018 paper by Rice, D.A., et al., which found that 21% of their respective sample suffered from PPP and identified preoperative knee pain intensity, trait anxiety, expected pain, and temporal summation as important predictive factors for PPP [11]. The presence of persistent pain following surgery can significantly impact the quality of life, serving as a crucial determinant of patient dissatisfaction post-TKA and potentially necessitating revision surgery [11,12]. 

Understanding and effectively addressing the predictors of PPP are paramount for enhancing patient outcomes and guiding clinical practices. The primary objective of this study is to enhance our understanding of the predictive factors associated with persistent postoperative pain (PPP) following total knee arthroplasty (TKA). By analyzing data from this retrospective study, the aim is to contribute broadly to the field, providing insights that can be applied to improve patient outcomes and clinical practices on a global scale, beyond a specific geographic region. This approach seeks to underscore the universal relevance and importance of identifying and addressing PPP in TKA patients.

## 2. Materials and Methods

### 2.1. Overview and Study Design

This is a retrospective analytic study that was conducted to identify predictors of persistent pain after total knee arthroplasty (TKA). The research was carried out at King Abdulaziz Medical City in Jeddah, Saudi Arabia, utilizing a comprehensive review of patient medical records through the “BestCare” system (internal automated medical records) to collect relevant data on postoperative outcomes and associated factors.

### 2.2. Eligibility Criteria

The study included all patients who underwent unilateral or bilateral TKA at King Abdulaziz Medical City between June 2016 and December 2023. A total of 838 patients were included. Inclusion criteria encompassed all adult patients (aged > 18 years) of both genders who received TKA. Exclusion criteria were applied to patients diagnosed with Raynaud’s syndrome or neurological diseases that affect sensation, as these conditions could confound the assessment of postoperative pain. 

### 2.3. Data Collection and Sources

Data were collected through a detailed review of “BestCare” electronic medical records by trained medical students under the supervision of an orthopedic surgeon. We conducted this study using a consecutive sampling technique for all eligible patients. The data abstraction process focused on various critical variables to understand postoperative pain outcomes. The collected data included the following:

- Demographic information: Age, gender, and body mass index (BMI), which is a good proxy for assessing overall body fat. For adults, normal weight is defined as a BMI of 18.5 to 24.9; overweight is defined as a BMI of 25.0 to 29.9; and obesity is defined as a BMI of ≥30.
- Comorbidities: Hypertension (HTN), diabetes mellitus (DM), obesity, tobacco use, pulmonary disease, cardiovascular disease, chronic kidney disease, autoimmune diseases, lung diseases, history of lower limb fractures, anxiety disorder, mood disorder, the American Society of Anesthesiologists (ASA) physical status classification system, and past surgeries.
- Clinical presentation: Reason for surgery, preoperative pain severity, preoperative quality of life, preoperative range of motion, preoperative pain duration, and length of hospital stays.
- Surgical details: Type of surgery, duration of surgery, date of surgery, intraoperative transfusion, blood loss quantity, whether the surgery was unilateral or bilateral, patella resurfacing, and tourniquet use and duration.
- Postoperative outcomes and complications: Infection, breakthrough pain, postoperative pain, postoperative pain duration, rehabilitation, thromboembolism, postoperative range of motion, and postoperative function.

### 2.4. Data Management and Analysis

The collected data were entered into an Excel 2021 spreadsheet (Microsoft Corporation, Redmond, WA, USA) and subsequently analyzed using JMP 17 software (JMP Statistical Discovery LLC, Cary, NC, USA). Continuous variables were summarized as means with standard deviations (SDs) or medians with interquartile ranges (IQRs) for non-normally distributed data, while categorical variables were described as percentages. We first performed a bivariate logistic analysis to assess the association between postoperative pain predictors (dependent variable) and each of the other variables described in the previous section (Section 2.3). This provided a preliminary understanding of how one explanatory variable may be associated with postoperative pain predictors without adjusting for other factors. Following this, we conducted multivariate logistic regression analyses to investigate the associations of the explanatory variables with postoperative pain predictors, while controlling for potential confounders or covariates. As none of the variables had a missing rate of >10%, all were included in the analysis. The results of the logistic regression analyses are presented as odds ratios (ORs) and 95% confidence intervals (CIs) that reflect the effect of each variable in our regression model. A *p*-value of <0.05 was considered statistically significant. Ethical approval was obtained from the relevant institutional review board, and patient confidentiality was strictly maintained throughout the study.

### 2.5. Ethical Considerations

This study was approved by King Abdullah International Medical Research Center (KAIMRC) with study number NRJ23J/235/09 (date: 30 October 2023). After the institutional review board (IRB) approval, the data were collected by authorized members.

## 3. Results

**Figure 1:**
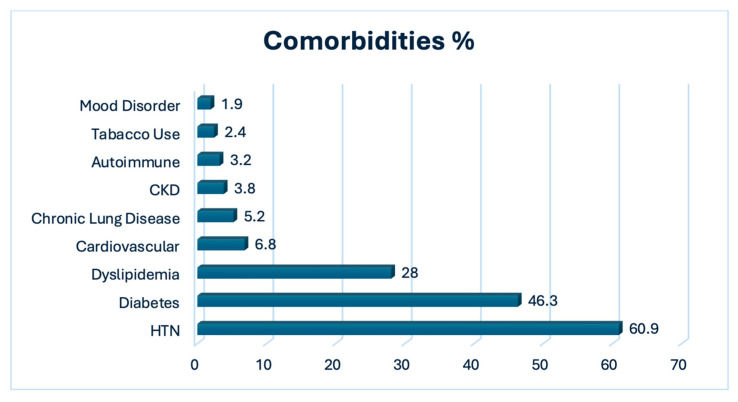
Comorbidities.

*Patients’ basic demographic information:* A total of 838 patients were included. The gender distribution shows that 28.5% (n = 239) of the patients were male, while 71.5% (n = 599) were female, indicating a higher proportion of females in this study. The mean age of patients was 65.4 years, with a standard deviation of 8.6 years, suggesting that most patients were between approximately 56.8 and 74 years old. Specifically, the age distribution shows that 3.34% of the patients were under 50 years old, 26.61% were between 51 and 60, 41.53% were between 61 and 70, and 28.52% were over 70 years. In terms of BMI, the majority of the patients included in this study (77.8%) were classified as obese (n = 652). A smaller proportion of the sample (17.3%) was overweight (n = 145) or of normal weight (4.1%) (n = 34) (Table 1). Figure 1 displays the prevalence of various comorbidities as percentages among this sample. The most common comorbidity is hypertension (HTN), affecting 60.9% of patients, followed by diabetes at 46.3%, dyslipidemia at 28%, and cardiovascular disease at 6.8%. Other less common conditions include chronic lung disease (5.2%), chronic kidney disease (ckd, 3.8%), autoimmune disorders (3.2%), tobacco use (2.4%), and mood disorder (1.9%).

*Clinical presentations: *Table 2 provides descriptive statistics for the clinical presentations of patients undergoing surgery. The primary reason for surgery among patients was osteoarthritis, accounting for 98.3% (824 patients), with only 1.7% (14 patients) undergoing surgery for other reasons. The average preoperative pain score was 3.4 ± 2.3, indicating a moderate level of pain across the group, although the standard deviation suggests that there was considerable variation in individual pain experiences. In terms of preoperative functional quality of life, 53.8% (344 patients) reported having normal functionality, while 33.8% (216 patients) required assistance, and 12.4% (79 patients) were wheelchair-bound before surgery. Regarding the range of motion, 42.1% (265 patients) had normal movement, while 57.9% (365 patients) experienced limitations. Less than 25% of the data are missing in two clinical presentation variables: preoperative functional quality of life and preoperative range of motion. Moreover, the mean duration of pain before surgery was 6.2 ± 4.9 years, further emphasizing the long-term discomfort that these patients faced. Additionally, the American Society of Anesthesiologists (ASA) classifications revealed that most patients were classified as ASA II (77.8%), with smaller groups classified as ASA I (5.0%) or ASA III (17.2%). The median surgery duration was 131.5 min (IQR 94–190), and intraoperative blood loss had a median value of 200 mL (with an interquartile range of 200 to 300 mL).

Furthermore, Table 3 summarizes the descriptive statistics for the patients’ surgical procedures and postoperative outcomes. Most surgeries were unilateral (72.2%), while 27.8% were bilateral. A torniquet was used in 90.9% of cases, and patella resurfacing was performed in only 3.8% of patients. Postoperative persistent pain was reported by 20.2% of patients, while 79.8% did not have this complication. Postoperative wound infections were rare, affecting 2.6% of patients, and breakthrough pain occurred in 2.3%. The median duration of postoperative pain was 13 days (with an interquartile range of 5 to 24 days). The postoperative range of motion was normal in 86.0% of patients, with 14.0% experiencing limitations. In terms of postoperative function, 73.5% of the patients had normal functionality, while 21.6% needed assistance and 4.9% were wheelchair-bound. The majority of patients (94.3%) underwent postoperative rehabilitation. Postoperative thromboembolism was reported in 1.9% of cases, and there was a very low mortality rate of 0.4%, with 99.6% of patients surviving. Less than 13% of the values are missing in two variables related to surgery and complications: postoperative range of motion and postoperative function.

Table 4 presents the results of both the bivariate and multivariate analyses, showing the odds ratios (ORs), 95% confidence intervals (CIs), and *p*-values of different clinical and demographic factors associated with the PPP. The bivariate regression analysis identified dyslipidemia as a significant predictor of postoperative persistent pain (PPP), with an odds ratio (OR) of 1.40 (*p* = 0.042). This OR suggests that patients with dyslipidemia have a 40% higher likelihood of experiencing postoperative persistent pain compared to those without dyslipidemia. The multivariate analysis found a younger age to be a significant predictor (OR 0.979, 95% CI 0.967–0.991, *p* = 0.001). This indicates that with each one-year increase in age, the odds of this outcome decrease by approximately 2.1%, suggesting that a younger age is associated with a higher likelihood of the outcome (PPP).

Other factors such as gender (OR 1.17, *p* = 0.423), diabetes mellitus (OR 1.05, *p* = 0.762, hypertension (OR 1.10, *p* = 0.579), cardiovascular disease (OR 0.92, *p* = 0.813), anxiety disorder (OR 0.71, *p* = 0.665), mood disorder (OR 1.31, *p* = 0.627), tobacco use (OR 0.99, *p* = 0.985), chronic kidney disease (OR 0.91, *p* = 0.839), chronic lung diseases (OR 0.99, *p* = 0.977), thromboembolism (OR 1.33, *p* = 0.674), and the type of surgery (OR 1.01, *p* = 0.998) were not significant predictors. The most common comorbidities were HTN (60.9%), diabetes (46.3%), and dyslipidemia (28%). This summary encapsulates key demographic data, clinical presentations, surgical details, postoperative outcomes, and significant predictors of postoperative pain in patients undergoing TKA. 

## 4. Discussion

In this study, we aimed to enhance our understanding of the predictive factors associated with persistent postoperative pain (PPP) following total knee arthroplasty (TKA). We found that a younger age and dyslipidemia were significant predictors of PPP following TKA. Dyslipidemia was associated with a 40% increase in the odds of developing PPP, while younger patients were more likely to experience PPP. Other factors, including gender, diabetes, hypertension, cardiovascular disease, anxiety, mood disorders, tobacco use, chronic kidney, and lung diseases, were not significant predictors.

This study had a disproportionate number of females compared to males. This could be explained by the fact that knee osteoarthritis is more common in females, likely due to hormonal factors and anatomical and kinematic differences in females [13]. Omar W. Althomali et al. [14] and Asim M. Makhdom et al. [15] also reported a higher prevalence in knee osteoarthritis in the female gender amongst the population of the Kingdom of Saudi Arabia. 

One of the key findings in this study is the identification of younger age as a significant predictor of PPP. This aligns with previous research suggesting that younger patients often report higher pain levels post-surgery, potentially due to higher physical activity expectations and demands [16]. This is similar to observations made by Jacqueline F. M. van Dijk et al. [17] and Jong-Ho Kim et al. [18] in their studies on predictors of postoperative pain after various surgeries (including total knee replacement). They reported that the maximum pain scores decreased significantly with age. Vahid Ashoorion et al. [19] carried out a systemic review and meta-analysis of predictors of persistent post-surgical pain following total knee arthroplasty and also reported an increased risk of persistent postoperative pain in patients in younger age groups. Additionally, dyslipidemia was found to be significantly associated with an increased risk of PPP in the bivariate analysis. Nevertheless, in Meert et al.’s study, the authors found that dyslipidemia was not a predictive value of PPP at six months [8]. Our findings contraindicate the current literature. We believe additional studies are needed to identify if there is any relationship between dyslipidemia and postoperative pain. 

This difference in findings between the bivariate and multivariate analyses highlights the importance of adjusting for potential confounders in a multivariate analysis. The bivariate analysis suggested a relationship between dyslipidemia and postoperative pain predictors when examined independently. However, the multivariate analysis suggests that the pain response may vary across age groups, potentially due to physiological or metabolic differences, recovery capacity, or pain perception in older versus younger individuals.

Moreover, gender was found to be an insignificant predictor of PPP in the bivariate analysis. However, a Ritter et al. study showed that the postoperative pain score was better in men compared to women (*p* < 0.0001) [20]. This discrepancy may be due to the imbalance in our gender sample, where the proportion of females is much higher than that of males (71.5% vs. 28.5%). This gender ratio imbalance may have influenced the results, as a larger female sample may skew the findings. We believe that a more balanced gender distribution in future studies could provide a clearer understanding of gender differences in pain perception.

Furthermore, in this study, it was found that BMI has no role in predicting PPP, similarly to Collins et al.’s study, which showed no difference in pain and function 24 months postoperatively across different BMI groups [21], although K Giesinger et al. [22] and Jamie E. Collins et al. [21] reported that an increased BMI is a significant predictor for postoperative pain following total knee arthroplasty. Contrary to Olsen et al.’s systematic review, we did not find anxiety and mood disorders to be significant predictors of PPP. Olsen et al. found mental health (anxiety, depression, psychological distress) to be one of the most important factors to predict pain 12 months postoperatively [23]. Other factors such as diabetes mellitus, hypertension, cardiovascular disease, chronic kidney disease, chronic lung disease, and tobacco use were not found to be significant predictors in the multivariate analysis. This suggests that while these factors may influence general health and recovery, they do not independently predict persistent pain following TKA in the studied population. 

Understanding the predictors of PPP is crucial for preoperative patient counseling and postoperative pain management strategies. The findings emphasize the need for tailored pain management plans, particularly for younger patients and those with dyslipidemia. By addressing these factors, healthcare providers can potentially reduce the incidence and severity of PPP, thereby enhancing patient satisfaction and overall outcomes.

The retrospective nature of this study inherently carries potential limitations, as it relies on pre-existing data, which may introduce biases related to the accuracy and completeness of records. In this study, we noticed some missing values in some variables, which may have influenced the findings. Additionally, this study was conducted at a single medical center, which may limit the generalizability of the findings to other populations. Therefore, it is possible that the associations observed between metabolic disease, age, and postoperative pain (PPP) in this study may not be directly translatable to different populations or healthcare environments. 

To address these limitations, future research should aim to validate these findings through prospective multicenter studies that actively collect data from a diverse patient population. Prospective studies would allow for more rigorous data collection, minimizing biases by ensuring that all relevant variables are systematically recorded and verified. Conducting multicenter studies would also enhance the diversity of the patient population, including various patient characteristics from different medical centers. Such studies would allow researchers to better evaluate the external validity of these findings and determine whether the observed associations hold true across different clinical settings and demographic groups.

## 5. Conclusions

This study identifies younger age and dyslipidemia as significant predictors of persistent postoperative pain following total knee arthroplasty. These findings suggest that demographic variables can play a crucial role in postoperative outcomes. Moreover, the findings of this study highlight dyslipidemia and young age as potential factors that can be considered in the personalized pain management strategies of TKA patients. The findings also highlight the importance of personalized pain management strategies and underscore the need for further research to explore the specific mechanism by which age and dyslipidemia contribute to persistent pain after TKA. Therefore, by improving our understanding of younger age and dyslipidemia as significant predictors of persistent postoperative pain following TKA, we can optimize the surgical outcomes and mitigate the development of persistent pain after TKA for those undergoing this common orthopedic procedure.

## Figures and Tables

**Table 1:** Patients’ basic demographic information.

Variables	N (%)
**Gender**	
Male	239 (28.5)
Female	599 (71.5)
**Age Distribution**	
<50	28 (3.34)
Between 51 and 60	223 (26.61)
Between 61 and 70	348 (41.53)
>70	239 (28.52)
Age (mean ± SD)	65.4 ± 8.6
**Body Mass Index**	
Normal weight	34 (4.1)
Overweight	145 (17.4)
Obese	652 (78.5)

**Table 2:** Clinical presentations.

Variable	N (%)
**Reason for surgery**	
Osteoarthritis	824 (98.3)
Other	14 (1.7)
Preoperative pain score ^1^	3.4 ± 2.3
Preoperative functional quality of life	
Normal	344 (53.8)
Needs assistance	216 (33.8)
In wheelchair	79 (12.4)
Preoperative range of motion	
Normal	265 (42.1)
Limited	365 (57.9)
Pain duration pre-surgery ^1^	6.2 ± 4.9
**ASA**	
ASA I	42 (5.0)
ASA II	652 (77.8)
ASA III	144 (17.2)
Surgery duration ^2^	131.5 (94, 190)
Intraoperative blood loss (mL) ^2^	200 (200, 300)

^1^ Mean ± SD. ^2^ Median (IQR).

**Table 3:** Surgery and complications.

Variable	N (%)
**Surgery type**	
Unilateral	605 (72.2)
Bilateral	233 (27.8)
**Torniquet**	
Yes	762 (90.9)
No	76 (9.1)
**Patella resurfacing**	
Yes	32 (3.8)
No	806 (96.2)
**Postoperative persistent pain**	
Yes	169 (20.2)
No	669 (79.8)
**Postoperative wound infection**	
Yes	22 (2.6)
No	816 (97.4)
**Postoperative breakthrough pain**	
Yes	20 (2.3)
No	818 (97.7)
Postoperative pain duration ^1^	13 (5, 24)
**Postoperative range of motion**	
Normal	637 (86.0)
Limited	104 (14.0)
**Postoperative function**	
Normal	483 (73.5)
Needs assistance	142 (21.6)
In wheelchair	32 (4.9)
**Postoperative rehabilitation**	
Yes	790 (94.3)
No	48 (5.7)
**Postoperative thromboembolism**	
Yes	16 (1.9)
No	822 (98.1)
**Death**	
Yes	3 (0.4)
No	835 (99.6)

^1^ Median (IQR).

**Table 4:** Comparative analysis of predictors for PPP.

Variable	Bivariate Analysis *		Multivariate Analysis **			
	OR	*p*-Value	OR	95% CI of OR		*p*-Value
				Lower	Upper	
Gender	1.17	0.423	1.078	0.709	1.639	0.727
Age	0.94	0.949	0.979	0.967	0.991	0.001
Diabetes mellitus	1.05	0.762	1.013	0.697	1.474	0.945
Hypertension	1.10	0.579	1.193	0.808	1.762	0.374
Cardiovascular	0.92	0.813	0.944	0.468	1.904	0.872
Anxiety disorder	0.71	0.665	0.734	0.145	3.710	0.708
Mood disorder	1.33	0.627	1.258	0.371	4.260	0.712
Dyslipidemia	1.40	0.042	1.370	0.937	2.005	0.104
Tobacco use	0.99	0.985	0.982	0.315	3.065	0.975
Kidney disease	0.91	0.839	0.852	0.336	2.160	0.735
Pulmonary disease	0.99	0.977	1.016	0.474	2.178	0.968
Body mass index	0.87	0.789	0.994	0.969	1.020	0.646
Gout	0.80	0.314	1.013	0.967	1.302	0.587
Thromboembolism	1.33	0.674	1.622	0.418	6.293	0.484
Previous knee procedure	1.13	0.754	0.846	0.544	1.315	0.457
History of lower limb fracture	0.49	0.496	0.430	0.053	3.512	0.431
Autoimmune disease	1.70	0.213	1.609	0.682	3.796	0.277
Surgery type	1.01	0.998	0.926	0.619	1.384	0.707

Note: CI = confidence interval and OR = odds ratio for the coefficients; * bivariate unadjusted odds ratio analysis; ** multivariate logistic regression adjusted odds ratio analysis.

## Data Availability

Dataset is available on request from the authors. The raw data supporting the conclusions of this article will be made available by the authors on request.
